# An ultra-compact angstrom-scale displacement sensor with large measurement range based on wavelength modulation

**DOI:** 10.1515/nanoph-2021-0754

**Published:** 2022-02-03

**Authors:** Yi Xu, Baowei Gao, Axin He, Tongzhou Zhang, Jiasen Zhang

**Affiliations:** State Key Laboratory for Artificial Microstructures and Mesoscopic Physics, School of Physics, Peking University, Beijing 100871, China; Institute of Navigation and Control Technology, China North Industries Group Corporation, Beijing 100089, China; Peking University Yangtze Delta Institute of Optoelectronics, Nantong 226010, Jiangsu, China

**Keywords:** displacement sensor, optical slot antennas, surface plasmon polaritons

## Abstract

Optical displacement metrology is important in nanotechnology and used to identify positions and displacements of nanodevices. Although several methods have been proposed, a sensor with ultracompact size, angstrom-scale resolution, and large measurement range is still lacking. We propose an optical displacement sensor with wavelength modulation that can demonstrate subwavelength footprint and angstrom-level resolution with large measurement range in this study. The proposed sensor consists of two optical slot antennas. Surface plasmon polaritons (SPPs) are launched at antennas and interfere when a tightly focused broadband light source illuminates the sensor. Spectrum of output SPPs presents a dip, which depends on the position of focal spot of incident light and is used to extract displacement. A maximum resolution of 0.734 nm was obtained. Furthermore, we used interference fringe of two broadband beams as light source and the measurement range of the sensor is not limited by the size of the tightly focused light source while maintaining high resolution. The method utilizes a new mechanism of wavelength modulation to overcome the trade-off between the high resolution and large measurement range, and achieve a variety of potential applications for nanometrology in the future.

## 1 Introduction

Integration of nanodevices in nanotechnology and manufacturing has progressed considerably in recent years [1]. Identifying positions and displacements of components with high precision and without destruction is crucial in functional nanosystems. The ability of optical metrology to measure displacement in a noncontact manner is an important advantage [2]. A variety of sensing configurations, including interferometer [3], [4], [5], [6], [7], [8], [9], quantum cascade laser [10], and optical metasurfaces [11, 12], have been used with the development of optical metrology to increase the resolution to the nanometric scale. However, the limited practical application of optical metrology due to large footprints fails to meet the demand of large-scale integration.

Surface plasmon polaritons (SPPs) originate from collective oscillations of conductive carriers and present the unique optical characterization of subwavelength confinement [13], [14], [15], [16], [17]. This property allows the use of metallic nanostructures in optical sensors for dimension minimization. Symmetrical antenna arrays are illuminated by inhomogeneous field at a specific wavelength and the intensity distribution of scattered light depends on the relative position of antennas and light source in these configurations [18]. The displacement can be retrieved by detecting the variation of the scattering intensity distribution. On the basis of this principle, Neugebauer et al. [19] experimentally demonstrated a displacement sensor composed of a single spherical gold nanoparticle under the illumination of collimated radially polarized beam with nanometer-scale resolution. In addition, a similar device with two identical rectangular plasmonic antennas with nanoscale dimensions was also investigated [20]. The nanosensor resolution was 3.3 nm in the deterministic measurement when high-order Hermite–Gaussian mode beam was subsequently used [21]. However, dependence of the resolution on the tight focusing of the incident beam limits the measurement range to around 100 nm in these proposed schemes. Therefore, a new sensing mechanism is needed because a displacement sensor with small footprint, angstrom-level resolution, and large measurement range is difficult to achieve but urgently necessary.

We propose a wavelength modulation sensing technique based on asymmetric plasmonic antenna pairs in this work to realize displacement sensors with characteristics of subwavelength dimensions, angstrom-level resolution, and large measurement range. The sensor is composed of two optical slot antennas with different dimensions. SPPs are launched at antennas when antennas are illuminated by a tightly focused broadband light source. The two SPP waves excited at antennas propagate and interfere. Output SPPs destructively interfere at a specific wavelength, that is, extinction wavelength depends on the incident position of the light source and the geometry design of antennas. The displacement between the antenna pair and the illumination field can then be acquired by measuring the extinction wavelength. A maximum resolution of 0.743 nm was obtained with a sensitivity of 0.455 nm/nm in the deterministic measurement of the experiment. We used the interference fringe of two broadband beams as the light source, and the extinction wavelength depends on the position and dispersion of the interference fringe to obtain a large measurement range. The measurement range of the sensor is not limited by the size of the tightly focused light source and high resolution is maintained. Furthermore, the measurement range can be further increased to satisfy engineering applications by adjusting the optical path difference. The proposed sensor with a new mechanism overcomes the trade-off between the high resolution and large measurement range and allows for its practical integration and application in fields of super-resolution microscopy [22, 23], semiconductor chip manufacture [24], single-molecule tracking [23, 25, 26], and nano-optomechanical systems.

## 2 Results

### 2.1 Design of the sensor

**Figure 1:**
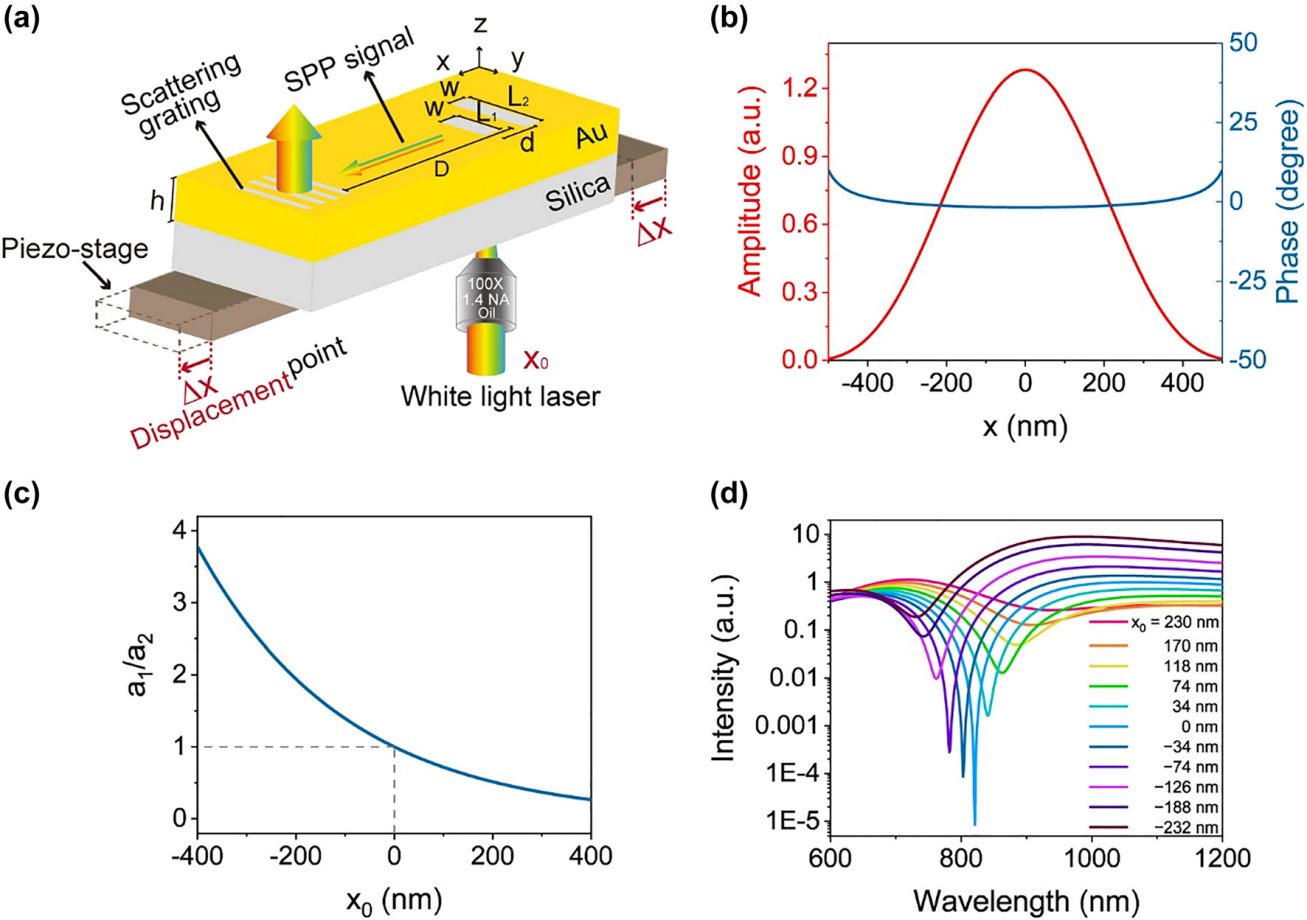
Working principles of the nanoscale displacement sensor. (a) Schematic of the nanoscale displacement sensor. (b) Calculated amplitude and phase distribution of a tightly focused light beam at *λ* = 815 nm. (c) Calculated $a_{1}/a_{2}$ versus *x*_0_ for a tightly focused light beam at *λ* = 815 nm. (d) Calculated output spectra for different *x*_0_ values. *ν*, *γ*_1_, and *γ*_2_ are set to be 0.2, 0.4, and 0.5, respectively. The resonant wavelengths of two antennas are set to be 889 and 727 nm, respectively.

The schematic of the proposed nanoscale displacement sensor is depicted in Figure 1(a). The sensor fabricated in an Au film with a thickness of *h* on a silica substrate is composed of two slot antennas with the same width (*w*) and different lengths (*L*_1_ and *L*_2_). The distance between the two antennas is *d*. The origin of the Cartesian coordinate is located at the center point between the two antennas at the air–Au film interface.

SPPs are launched at the two antennas and propagate along the *x*-axis at the air–Au film interface. The two antennas can be considered two SPP point sources when an *x*-polarized and tightly focused broadband light beam is normally incident from the substrate side along the +*z*-axis. The two launched SPP waves can interfere with each other. For a certain detection point at a distance of *D* (*D* ≫ *d*) from the origin along the +*x*-axis, the intensity of propagating SPPs at the wavelength of *λ* can be expressed as follows:

$$I\left(\lambda ,\;x_{0}\right)=|C_{1}E_{1}\left(\lambda ,\;x_{0}\right)\text{exp}\left[i\left(\frac{2\pi n_{\text{eff}}}{\lambda }\left(D-\frac{d}{2}\right)+\varphi_{1}\left(\lambda ,\;x_{0}\right)\right)\right]$$

(1) $$\begin{array}{c} +C_{2}E_{2}\left(\lambda ,\;x_{0}\right)\text{exp}\left[i\left(\frac{2\pi n_{\text{eff}}}{\lambda }\left(D+\frac{d}{2}\right)+\varphi_{2}\left(\lambda ,x_{0}\right)\right)\right]{|}^{2} \end{array}$$

where *E*_1_ and *E*_2_ represent initial amplitudes of the electric field of SPPs at the center of the two antennas in the air–Au interface; *φ*_1_ and *φ*_2_ are corresponding initial phases; *C*_1_ and *C*_2_ represent decay parameters for SPPs propagating from antennas to the detected point; *n*_eff_ is the effective refractive index of SPPs at the air–Au film interface; *x*_0_ is the coordinate of the focal spot of the incident beam; and *E*_1_, *E*_2_, *φ*_1_, and *φ*_2_ are functions of *x*_0_ and *λ*, thereby indicating that the output spectrum depends on the position of the incident beam. If *C*_1_ *E*_1_ ≈ *C*_2_ *E*_2_ and total phase difference $\Delta \varphi =\varphi_{2}-\varphi_{1}+\frac{2\pi n_{\text{eff}}}{\lambda }d\approx \pi$ are satisfied at a wavelength for a fixed *x*_0_, then destructive interference occurs and a dip will appear in the spectrum at a specific wavelength, that is, the extinction wavelength.

Amplitudes and phases of the two SPP waves change and result in the shift of the extinction wavelength when the focal spot moves. We calculated the movement of output spectra with respect to the position of the tightly focused light source using a semi-analytical method to illustrate this scenario further. Electromagnetic coupling between them should be considered given that the two slot antennas are placed close to each other. The two antennas function as two dipole resonators and coupled harmonic oscillator theory is utilized to simplify the analysis in this study. The two oscillators present eigen-frequencies of *ω*_1_ and *ω*_2_. The amplitude and phase distributions of the focal spot at *λ* = 815 nm focused by an objective with a numerical aperture (NA) of 1.4 are shown in Figure 1(b). The results showed that the phase slightly changes. We neglect the phase change of the light source and amplitudes of excitation fields illuminated on the two antennas are *a*_1_ and *a*_2_ in the analysis. Amplitudes of electric fields of the two oscillators can be expressed as follows:

(2) $$\begin{array}{c} \left\{ \begin{array}{c} {\ddot{E}}_{1}\left(t\right)+\gamma_{1}{\dot{E}}_{1}\left(t\right)+\omega_{1}^{2}E_{1}\left(t\right)+\nu E_{2}\left(t\right)=a_{1}{\text{e}}^{i\omega t}\\ {\ddot{E}}_{2}\left(t\right)+\gamma_{2}{\dot{E}}_{2}\left(t\right)+\omega_{2}^{2}E_{2}\left(t\right)+\nu E_{1}\left(t\right)=a_{2}{\text{e}}^{i\omega t} \end{array}\right. \end{array}$$

where *γ*_1_ and *γ*_2_ represent damping parameters, *ν* is the coupling coefficient between these two resonators. The electromagnetic response can be expressed as follows:

(3) $$\begin{array}{c} \left\{ \begin{array}{c} E_{1}\left(t\right)=c_{1}\left(\omega \right){\text{e}}^{i\omega t}\\ E_{2}\left(t\right)=c_{2}\left(\omega \right){\text{e}}^{i\omega t} \end{array}\right. \end{array}$$

where $c_{1}\left(\omega \right)$ and $c_{2}\left(\omega \right)$ represent the complex amplitude of the two resonators and can be expressed as:

(4) $$\begin{array}{c} \left\{ \begin{array}{c} c_{1}\left(\omega \right)=\frac{\left(\omega_{2}^{2}-\omega^{2}+i\gamma_{2}\omega \right)a_{1}-\nu a_{2}}{\left(\omega_{1}^{2}-\omega^{2}+i\gamma_{1}\omega \right)\left(\omega_{2}^{2}-\omega^{2}+i\gamma_{2}\omega \right)-\nu^{2}}\\ c_{2}\left(\omega \right)=\frac{\left(\omega_{1}^{2}-\omega^{2}+i\gamma_{1}\omega \right)a_{2}-\nu a_{1}}{\left(\omega_{1}^{2}-\omega^{2}+i\gamma_{1}\omega \right)\left(\omega_{2}^{2}-\omega^{2}+i\gamma_{2}\omega \right)-\nu^{2}} \end{array}\right. \end{array}$$

The calculated $a_{1}/a_{2}$ at *λ* = 815 nm with respect to the position of the focal spot when the light source moves along the *x*-axis is shown in Figure 1(c). The output spectrum can be calculated using $I\left(\omega \right)={\left|c_{1}\left(\omega \right)+c_{2}\left(\omega \right)\cdot \text{exp}\left(i\Delta \theta \right)\right|}^{2}$ , where Δθ is the phase retardation induced by the spatial separation between two antennas. The output spectrum *I* for varied *x*_0_ is calculated and the result is plotted in Figure 1(d), in which the excitation wavelength shifts with the movement of sensor. As a result, the displacement can be acquired from the extinction wavelength.

**Figure 2:**
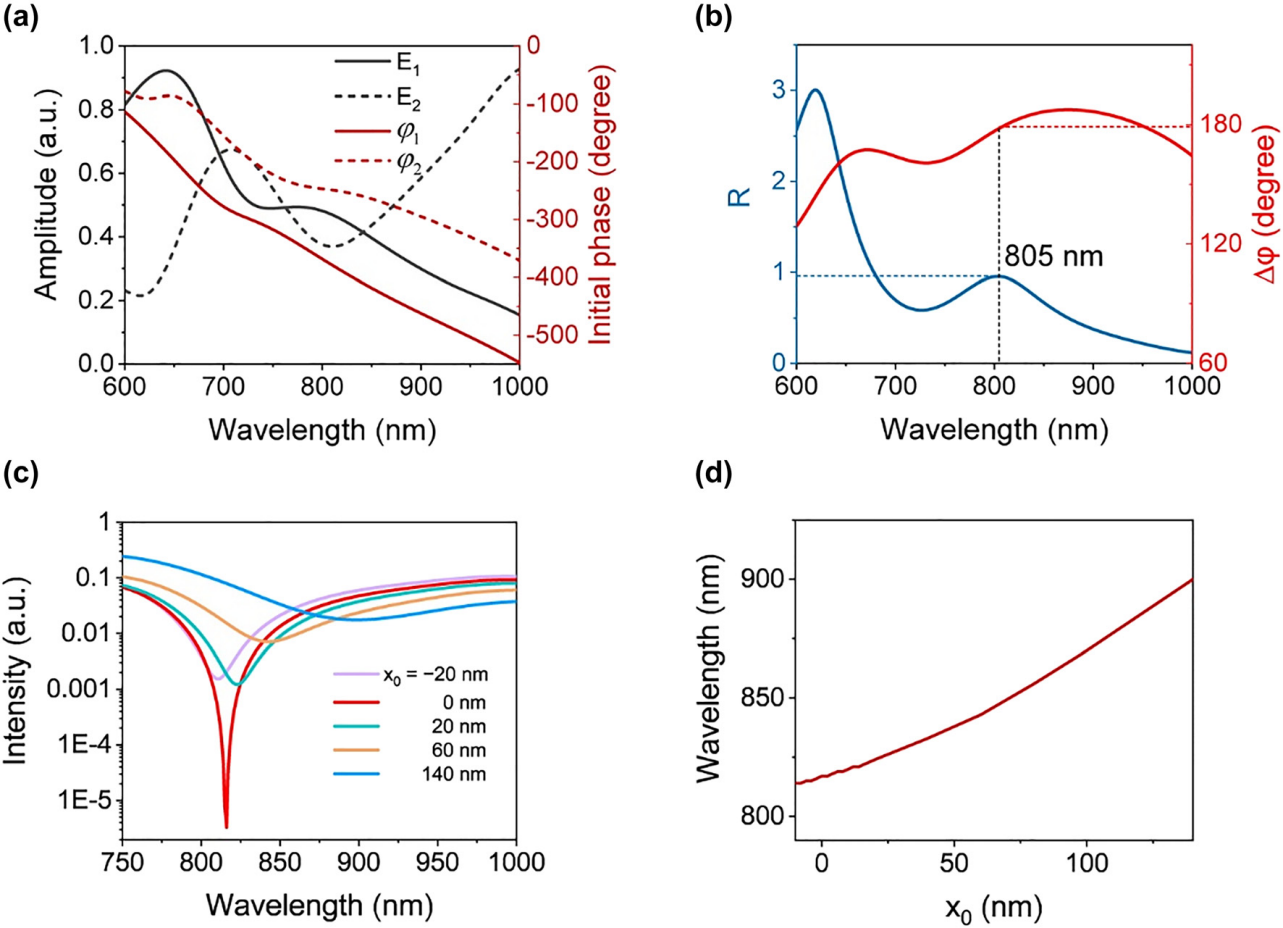
Calculation results of the nanoscale displacement sensor. (a) Calculated electric field amplitudes (solid lines) and the initial phase (dashed lines) at centers of the two antennas in the *xy* plane versus the wavelength. (b) Calculated amplitude ratio of SPPs launched by the two antennas (blue line) and Δ*φ* (red line) versus the wavelength for *d* = 120 nm. (c) Calculated output spectra for various displacements. (d) Calculated extinction wavelength versus the displacement.

We used a finite-difference time-domain (FDTD) method to design the parameter of the sensor. A broadband light beam was focused on the two antennas at the silica–Au interface using an objective lens with an NA of 1.4 in the simulation. The amplitude and phase distributions at the silica–Au film interface are presented in Figure 1(b). Geometry parameters were optimized at *h* = 200 nm, *L*_1_ = 190 nm, *L*_2_ = 300 nm, *w* = 70 nm, and *d* = 120 nm to satisfy conditions of destructive interference. Different lengths of antennas were designed to adjust initial phases and intensities of launched SPPs. The distance *d* was optimized to satisfy the phase condition of the destructive interference at the extinction wavelength. We calculated amplitudes (*E*_1_ and *E*_2_) of the electric field at centers of antennas with respect to the wavelength using the FDTD method when *x*_0_ = 0. The results are plotted in Figure 2(a). Corresponding initial phases $\varphi_{1}$ and $\varphi_{2}$ of the two antennas are also plotted with dashed lines in Figure 2(a). The calculated amplitude ratio *R* = *C*_1_ *E*_1_/*C*_2_ *E*_2_ and the total phase difference Δ*φ* are illustrated in Figure 2(b) (*C*_1_ and *C*_2_ are calculated in Supplementary material Section 1). At the wavelength of *λ* = 805 nm, *R* = 0.96, and Δ*φ* = 178.2°, which lead to a sharp dip. The red line in Figure 2(c) illustrates the calculated intensity of propagating SPPs launched by the antenna pair. The spectrum shows the minimum value at 815 nm, which is the extinction wavelength that slightly deviates from the design wavelength of 805 nm. The deviation originates from the intensity and phase change of SPPs launched by the right antenna when they pass over the left antenna during propagation.

When *x*_0_ changes, the calculated amplitude ratio and Δ*φ* change simultaneously (see details in Supplementary material Section 2), which influences the destructive interference and causes a shift in the extinction wavelength. Calculated output spectra with different *x*_0_ values are illustrated in Figure 2(c). The spectra are presented in logarithmic coordinates to highlight the extinction wavelength. The spectral dips have similar broadening for different *x*_0_ (see Supplementary material Section 3). The calculated extinction wavelengths are obtained by extracting the wavelengths with the minimum intensity values of the calculated spectra. The extinction wavelength with respect to *x*_0_ shown in Figure 2(d) is a monotonic function of *x*_0_ that can be used to obtain the value of *x*_0_. The wavelength sensitivity (d*λ*/d*x*_0_) reaches 0.628 nm/nm on average; hence, the wavelength shifts by 0.628 nm for each displacement of 1 nm.

### 2.2 Fabrication and experiment

**Figure 3:**
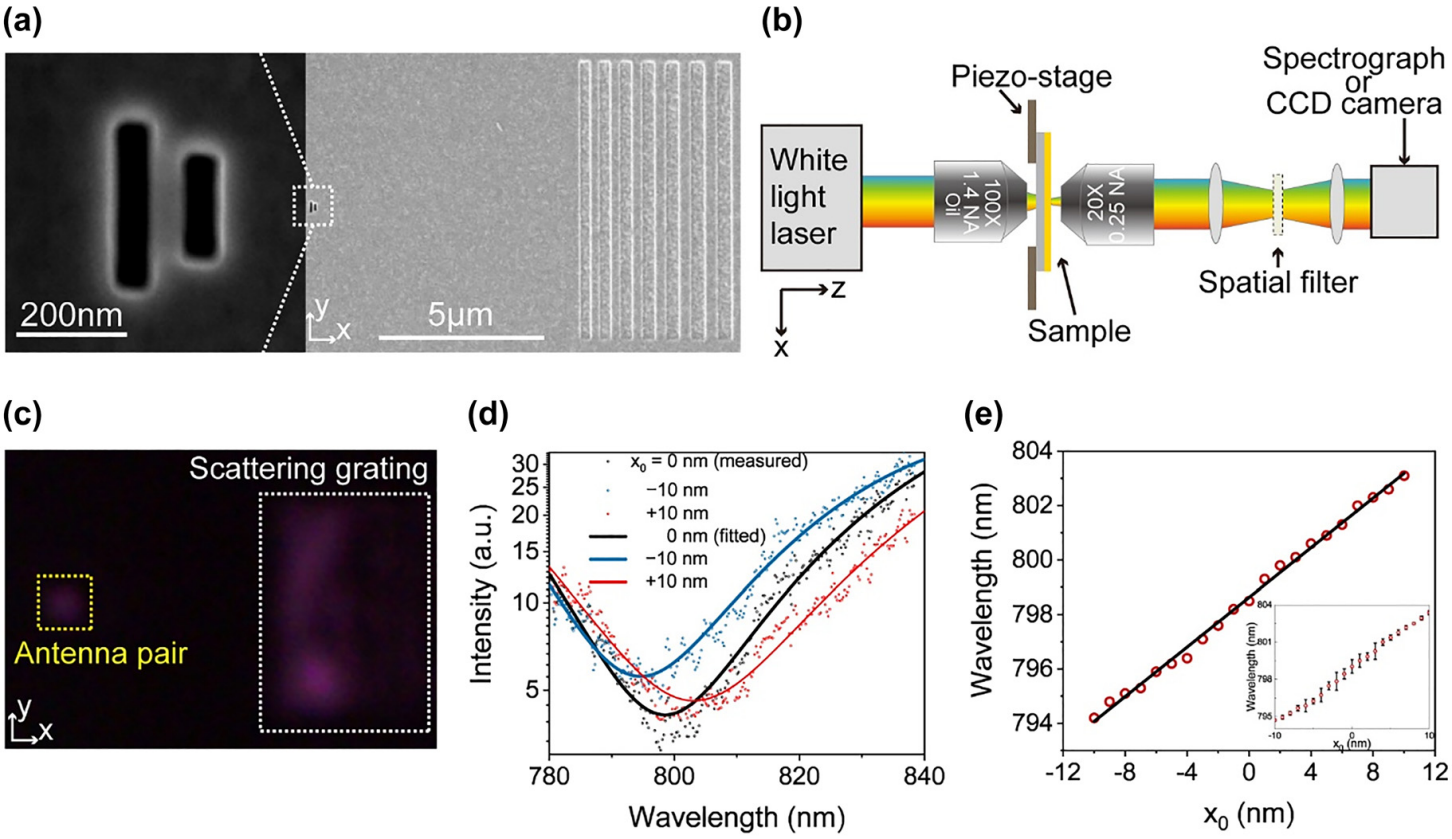
Experimental results of the sensor. (a) SEM image of fabricated slot antennas and scattering grating. Inset: Magnified SEM image of the antenna pair. (b) Schematic of the experimental setup. (c) CCD image of the antenna pair and diffraction grating under the illumination of an *x*-polarized supercontinuum light laser. (d) Output spectra (dots) and the corresponding polynomial fittings (lines) for different *x*_0_ values. (e) Extinction wavelength versus the displacement. The line presents the linear fitting. Inset: Extinction wavelength and measurement uncertainty versus the displacement.

The device was fabricated through deposition in a 200 nm-thick Au film on a silica substrate, followed by slot antenna etching based on focused ion beam (FIB) milling for experimental demonstration. A scanning electron microscopy (SEM) image of the sensor is shown in Figure 3(a). The geometric parameters of antennas are consistent with designed parameters. Chirped grating was fabricated 8 μm away from the antenna pair for the measurement of the output spectrum. The grating with a duty cycle of 0.5 and period varying from 526 to 800 nm can be used as a broadband output coupler with a 318 nm-wide working range (additional details are presented in Supplementary material S4).

A sketch of the experimental setup is illustrated in Figure 3(b). The sensor is mounted on a piezo stage (P-611.3, Physik Instrumente, Germany) with a minimum step of 1 nm, and the lateral displacement between the antenna and the light source is controlled by moving the stage along the *x*-axis. An *x*-polarized supercontinuum laser beam (SC-5, YSL Photonics, China) was focused using an oil-immersion objective (100×, NA = 1.4) and normally illuminated upon the antenna pair from the silica substrate along the +*z*-axis. The spectrum of the light source used in the experiment is shown in Figure S5(a) in Supplementary material. SPPs launched by the antenna pair propagate along the +*x*-axis and are scattered into free space via scattering grating. The scattered light is collected by another objective (20×, NA = 0.25) and coupled to a spectrograph (Zolix Omni-λ300i). The collection efficiency of the objective lens is calculated to be 37.3% (see more details in Supplementary material Section 5). In addition, a charge coupled device (CCD) camera is used to image the sensor.

Figure 3(c) shows the CCD image of antennas and scattering grating under the illumination of the light beam. The spot in the yellow dotted frame indicates the light directly transmitted from antennas, and the output signal scattered at the grating is marked by the white dotted frame. The directly transmitted light from the antenna pair is blocked using a spatial filter during measurement in the experiment.

The captured spectrum at *x*_0_ = 0 (black dots) in Figure 3(d) shows a dip at around 800 nm. A polynomial function was used to fit experimental data to characterize the dip, as shown by the black line in Figure 3(d). The wavelength of the minimum polynomial function was extracted as the experimental extinction wavelength. Here, the extinction wavelength is 798.5 nm for *x*_0_ = 0. Captured spectra and corresponding polynomial fittings for *x*_0_ values of −10 and 10 nm are also shown in Figure 3(d), which shows extinction wavelengths of 794.2 and 803.1 nm, respectively. The results indicated that output spectra and corresponding extinction wavelengths strongly depend on positions of the incident focal spot and can be used for displacement sensing. The output spectrum was recorded at each step when the piezo stage with a step of 1 nm was moved. The extinction wavelength is plotted in Figure 3(e). The results showed that the extinction wavelength monotonically increases with respect to the displacement. The black line denotes the linear fitting with a slope of *k* = 0.455 ± 0.005 nm/nm, which is the experimental sensitivity of the sensor. The fitting errors for each spectrum are present in Figure S6 in the Supplementary material, and the small errors indicate the reliability of the fitting (see details in Supplementary material Section 6). Output spectra were measured repeatedly at each step to obtain the determinist resolution of the sensor. The mean value and standard deviation of the extinction wavelength are presented in the inset of Figure 3(e). The results showed that the measurement error of the extinction wavelength is estimated at *σ*_r_ = 0.33 nm. Therefore, the resolution of the displacement is estimated at 0.743 ± 0.008 nm. The error in the measurement originates from the jitter of measured data mainly due to the vibration of experimental platform [21] and errors of polynomial fitting that limit the measurement resolution.

The results showed that the sensor can reach angstrom-level displacement resolution experimentally with wavelength modulation using a tightly focused light source. Considering the wavelength resolution of spectrographs, extreme sensing resolution can be stepped into the sub-angstrom level on the limit. However, the tightly focused light source limits the measurement range of the displacement. Hence, we will show the enlargement of the measurement range using a periodically dispersive light source while maintaining high resolution.

### 2.3 Design of the sensor with a dispersive interference fringe light source

**Figure 4:**
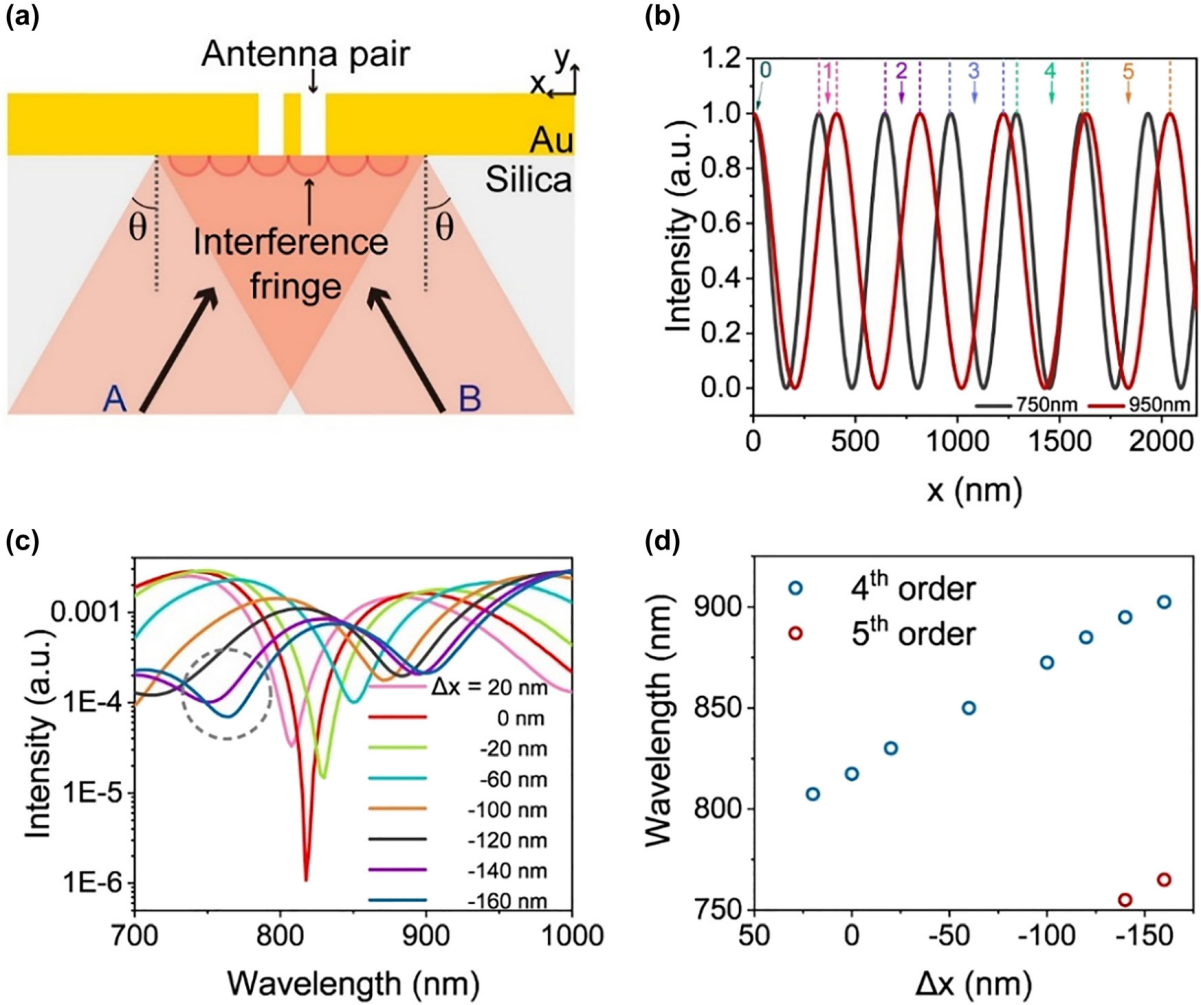
Calculation results of the sensor using the dispersive interference fringe light source. (a) Schematic of the dispersive interference fringe light source with an inclination angle of *θ*. (b) Calculated electric field intensity versus *x* for *λ* of 750 and 950 nm. The number refers to the interference order. (c) Calculated output spectra for various displacements. (d) Calculated extinction wavelength versus the displacement.

The measurement range of the displacement is usually negatively correlated with the resolution for the utilization of a tightly focused light source. The resolution improves while the measurement range decreases as the size of the light spot decreases. We design a periodically dispersive light source formed through broadband light interference to solve this trade-off and enlarge the measurement range while maintaining high resolution. As shown in Figure 4(a), two broadband light beams are incident upon the antenna pair with incident angles of *θ* and interfere. The period of the interference fringe is Δ=λ/(2n⋅sin θ) , where *n* is the refractive index of silica.

Figure 4(b) shows intensity distributions for *λ* of 750 and 950 nm and *θ* = 50° when the equal optical path position of the two beams is located at *x* = 0 at the silica–Au interface and intensities of the two broadband light beams are equal. The zero-order interference fringe at *x* = 0 demonstrates the absence of dispersion while its intensity distribution with respect to *x* is similar to that of a tightly focused light spot through a cylindrical lens. If the zero-order interference fringe is used as the light source of the sensor, then its sensing properties should be comparable with the aforementioned results with tightly focused light source in Figure 2(d). However, a large *θ* can result in a small full width at half maximum (FWHM) of the interference fringe and benefit the resolution. We set *θ* to 50° considering the NA of the objective used in the experiment.

We mainly analyze the effect of the dispersive interference fringe on the displacement sensing in this study. As shown in Figure 4(b), the intensity distribution of the light source with respect to *x* is a sine function for a specific wavelength. The maximum intensity of different wavelengths, except for the zero-order, is located at different spatial positions. The maximum intensity of the fourth-order dispersion fringe at the long wavelength begins to overlap spatially with that of the fifth order at the short wavelength in the working wavelength range of 750–950 nm. The destructive interference of SPPs occurs according to the design of the antenna pair when the maximum intensity at the special wavelength is located at *x* = 0. The spectrum of the output signal will then demonstrate a dip near this wavelength.

For example, we assume that the maximum intensity of the fourth interference fringe of the light *λ* = 815 nm is located at *x* = 0. The calculated output spectrum with a dip at 815 nm is shown in Figure 4(c). The calculated output spectra for different relative displacements Δ*x* when the interference fringe moves are also presented in Figure 4(c) in logarithmic coordinates (see details in Supplementary material S7). The extinction wavelength shifts with respect to the relative displacement. In addition, a new dip that appears in the spectrum at the short wavelength (dashed circle in Figure 4(c)) originates from the fifth-order interference fringe at the short wavelength when the displacement is Δ*x* = −140 nm. As a result, large displacement can be measured using the extinction wavelength of the fifth-order interference fringe. The measurement range can be enlarged effectively using additional orders of the interference fringe. The extinction wavelengths were obtained by extracting the wavelengths of the minimum values of the dips in the spectra. The extinction wavelength with respect to the displacement plotted in Figure 4(d) demonstrates the approximate linear relationship between the extinction wavelength and the displacement. Dual extinction wavelengths that appear in the part where the two periods intersect allow the sensor to demonstrate a large measurement range using several periods when the order number is larger than four.

### 2.4 Experimental results of the sensor with a dispersive interference fringe light source

**Figure 5:**
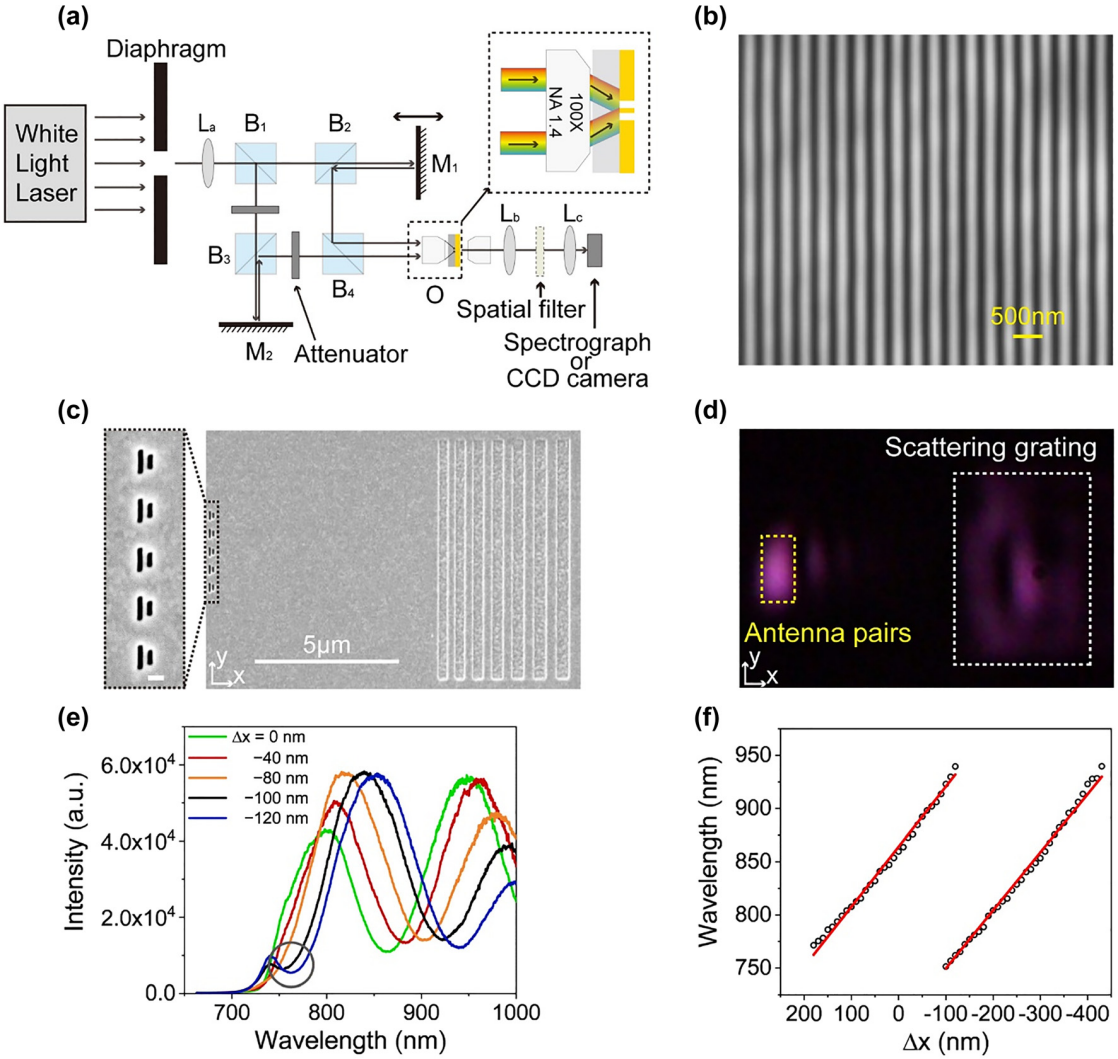
Experimental results of the sensor with a dispersive interference fringe light source. (a) Schematic of the experimental setup. (b) CCD image of the interference fringe when a narrowband filter of 810 nm is used. (c) SEM image of fabricated antenna pairs and the scattering grating. Inset: Magnified SEM image of antenna pairs. (d) CCD image of antenna pairs and diffraction grating under the illumination of an *x*-polarized supercontinuum laser beam. (e) Output spectra for different displacements Δ*x*. (f) Extinction wavelength versus the displacement Δ*x*. Red lines represent linear fittings.

The experimental setup is illustrated in Figure 5(a). Dispersive interference fringes are built up by the interference of two supercontinuum laser beams. A square diaphragm illuminated by the supercontinuum laser beam is imaged onto the sensor using an imaging system composed of a lens L_a_ and an oil-immersion objective O (100×, NA = 1.4). The light beam is separated into two paths using four beam splitter prisms (B_1_, B_2_, B_3_, and B_4_) and two mirrors (M_1_ and M_2_) between the lens and the objective. Mirror M_1_ is placed on the manual translation stage to adjust the optical path difference between the two paths. Intensities of the two paths are independently controlled using two attenuators for the desired intensity ratio.

As shown in the inset of Figure 5(a), two beams are incident onto the objective lens through the edge of the back focal plane. As a result, the two beams overlap and interfere at the silica–Au interface. Incident angles *θ* were set to ∼50° by adjusting incident positions of the two beams at the back focal plane of the objective lens. The output light signal is collected by an objective (20×, NA = 0.25) and two lenses (L_b_ and L_c_) and then coupled into a spectrograph or a CCD camera. A spatial filter is inserted to reduce stray light.

We inserted an 810 nm narrowband filter ahead of the objective O and imaged interference patterns on the CCD camera to verify the interference fringe, as shown in Figure 5(b). The intensity distribution of the fringe exhibits a period of 336 nm, which indicates *θ* = 53°.

The sample was fabricated on a 200 nm-thick Au film deposited on a silica substrate. Antenna pairs and scattering grating were etched via the FIB milling process. The SEM image is shown in Figure 5(c). Five antenna pairs along the *y*-axis were arranged with a period of 600 nm to improve the detected signal intensity given that the intensity distribution of the illuminated beam is constant along the *y*-axis. Chirped grating with parameters consistent with gratings in Section 2.3 was fabricated 8 μm away from antenna arrays to scatter propagating SPPs in the broadband range. Figure 5(d) shows the captured CCD image of the sample. A spatial filter was used to avoid the disturbance of the stray light scattered by antenna pairs and ensure that only the signal output scattered from the grating can be coupled into the spectrograph to improve the signal-to-noise ratio.

We used the fourth-order interference fringe to illuminate antenna pairs in the experiment. The measured spectra are shown in Figure 5(e), which are stronger and smoother than those in Figure 3(d) because five antenna pairs were used here. The extinction wavelengths were obtained from the smoothed data of experimental spectra shown in Figure S8 presented in Supplementary material. The green line of the spectrum presents an extinction dip at 862 nm, and the position of the interference fringe is defined as *x* = 0. We moved the sample and measured output spectra with respect to the displacement. The extinction wavelength shifts monotonically from 862 to 945 nm as the relative displacement increases from 0 to 120 nm. Another extinction dip emerges at 760 nm when the relative displacement is 100 nm, as indicated by the gray circle in Figure 5(e). The new extinction dip originates from the illumination of the fifth-order fringe and redshifts with the movement of the displacement. As a result, the extinction wavelength from the fifth-order fringe can be used for sensing when *x* > 120 nm. Several orders of the interference fringe can be used and the measurement range can be enlarged in the working wavelength range of 750–950 nm when extinction dips with different wavelengths caused by adjacent orders of the interference fringe simultaneously appear.

Figure 5(f) shows the experimental extinction wavelength with respect to the displacement in two periods. The linear fitting result shows sensitivities of the two orders of 0.565 and 0.543 nm/nm, which are comparable with the findings in Section 2.2 when the tightly focused light source is used. Although the FWHM of the interference fringe for a fixed wavelength is smaller than that of the tightly focused light source, their resolutions should be similar considering the stability of the experimental system, especially the resolution of 1 nm of the piezo stage. Although only two orders of the interference fringe were utilized in the experiment, additional periods can be used due to the appearance of the dual extinction wavelength in the part where the two periods intersect. Moreover, the position of the interference fringe can be also moved by changing the optical path difference. The same period can be used to implement sensing in a very large measurement range when the position of the interference fringe moves.

## 3 Discussion

Conventional displacement sensors use intensity distribution of the output light as the sensing signal. A trade-off exists between high resolution and large measurement range, that is, the measurement range decreases with the improvement of the resolution. We proposed a new mechanism of wavelength modulation, to implement displacement sensing in this work. We can regulate properties of the sensor by modulating both the intensity distribution and dispersion of the incident light source. A nondispersive light source was used and an angstrom-level resolution was obtained using the wavelength modulation in the first experiment. The sensor can achieve a large measurement range without loss of resolution by controlling the dispersion in the interference fringe of two broadband beams.

The period and FWHM of the interference fringe depend on the incident angle, which influences the resolution, sensitivity, and measurement range of the sensor simultaneously (see details in Supplementary material Section 9). A larger incident angle results in a smaller FWHM of the interference fringe, which benefits to the resolution of the sensor. Even for a smaller incident angle, the maximum intensity of the fourth-order dispersion fringe at the long wavelength begins to overlap spatially with that of the fifth-order at the short wavelength in the working wavelength range of 750–950 nm. Because the smaller the incident angle, the larger is the period of the interference fringe. Therefore, a smaller incident angle results in a larger measurement range and a smaller sensitivity.

Moreover, the interference fringe can also be moved by changing the optical path difference. Therefore, the measurement range can be only limited by the size of the incident beam when the interference fringe is moved by changing the optical path difference. The wavelength modulation provides increased flexibility in the design of the sensor light source. Dispersion elements can be used in the optical path to improve the performance of the sensor.

Apart from the design of the light source, the structure of antennas can still improve. Two antennas are regarded as two point sources for the acquisition of destructive interference in this work. Furthermore, antennas can be designed to act as multiple point sources to obtain high resolution and sensitivity. In addition, two-dimensional displacement sensing can be achieved by integrating two antenna pair arrays (see Supplementary material Section 10).

## 4 Conclusions

We established an optical displacement sensor to remove the trade-off between high resolution and large measurement range in existing displacement sensors. The proposed sensor based on the interference of an asymmetric nanoantenna pair can realize wavelength modulation with the change of displacement and support the illumination with both tightly focused and dispersive light sources. The sensor presents an angstrom-level resolution of 0.743 nm with the illumination of the tightly focused Gaussian beam. Replacing the source with a dispersive interference fringe enlarges the measurement range to the micrometer scale while maintaining the resolution at the same level. The proposed sensor will offer an ideal solution for modern nanometrology and contribute to the development of semiconductor lithography and super-resolution microscopy.

## Supplementary Material

Supplementary Material
