# Supplementary material for: An ultra-compact angstrom-scale displacement sensor with large measurement range based on wavelength modulation
Source: Nanophotonics. 2022 Feb 3;11(6):1167–76. doi: 10.1515/nanoph-2021-0754 (PMC11501363; doi:10.1515/nanoph-2021-0754)
Supplement: Supplementary file 1 — Supplementary Material [file j_nanoph-2021-0754_suppl.pdf]

Yi Xu‡, Baowei Gao‡, Axin He, Tongzhou Zhang, and Jiasen Zhang

Supplementary material for "An ultra-compact angstrom-scale displacement sensor with large measurement range based on wavelength modulation"

***Corresponding authors: Jiasen Zhang,** State Key Laboratory for Artificial Microstructures and Mesoscopic Physics, School of Physics, Peking University, Beijing, 100871, China. Peking University Yangtze Delta Institute of Optoelectronics, Nantong 226010, Jiangsu, China. E-mail: jszhang@pku.edu.cn. https://orcid.org/0000-0002-0751-6697
Yi Xu, Axin He, Tongzhou Zhang, State Key Laboratory for Artificial Microstructures and Mesoscopic Physics, School of Physics, Peking University, Beijing, 100871, China.
Baowei Gao, Institute of Navigation and Control Technology, China North Industries Group Corporation, Beijing, 100089, China.
‡These authors contributed equally.

Keywords: displacement sensor, optical slot antennas, surface plasmon polaritons

1. Calculated decay parameters C_1 and C_2

C_1 and C_2 are decay parameters for the SPPs propagating from the two antennas to the detection point, which are obtained at $x = +2$ μm in the simulation. The calculated decay parameters are shown in Figure S1.

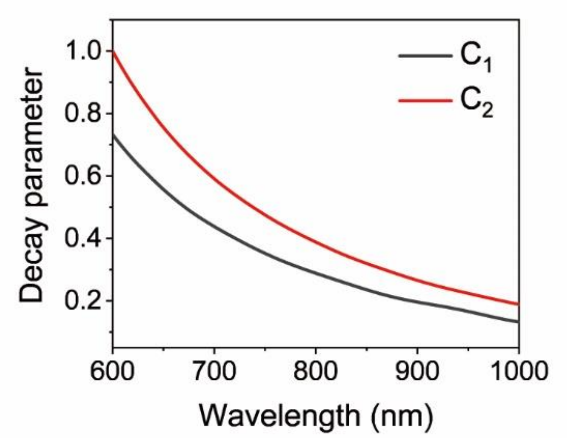

Figure S1: Calculated decay parameters C_1 and C_2 versus the wavelength.

2. Calculated amplitude ratio R and total phase difference $\Delta\varphi$ with respect to the wavelength for different x_0

The calculated amplitude ratio R and total phase difference $\Delta\varphi$ with respect to the wavelength for different x_0 are shown in Figures S2(a) and S2(b), respectively. It is seen that both the amplitude ratio R and the total phase difference $\Delta\varphi$ depend on the displacement x_0, which influences the interference and causes a shift in the extinction wavelength.

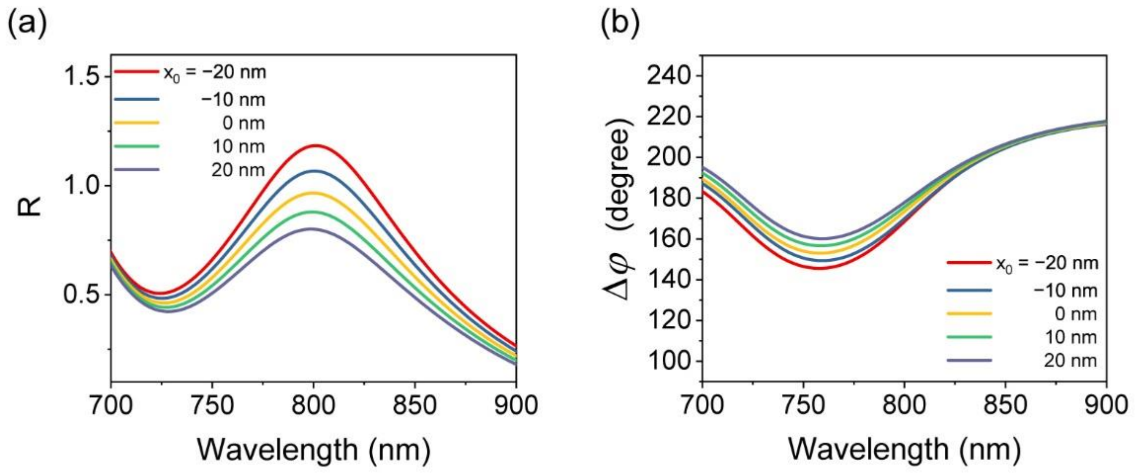

Figure S2: (a) Calculated amplitude ratio R of SPPs launched by the two antennas for various displacements x_0. (b) Calculated $\Delta\varphi$ versus the wavelength for different x_0.

3. Calculated output spectra in linear coordinates

The calculated output spectra with varied displacements in Figure 2(c) are plotted in logarithmic coordinates to show the wavelength shift more clearly. Here, we replot the spectra in linear coordinates to show the broadening of the spectra in Figure S3. The dips show similar broadening.

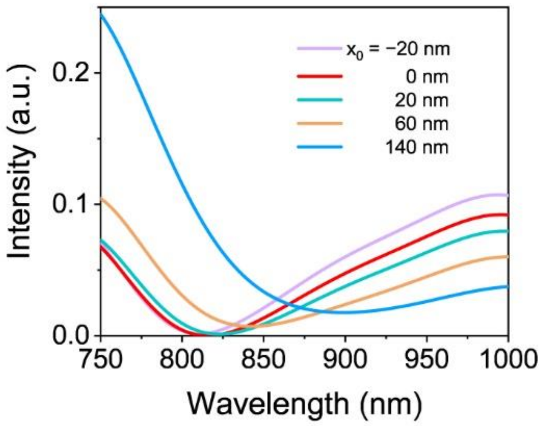

Figure S3: Calculated output spectra for various displacements under the illumination of the tightly focused beam in linear coordinate.

4. Diffraction efficiency of the scattering grating with respect to the wavelength

A chirped grating is used to obtain broadband diffraction. The grating has a duty cycle of 0.5 with the period varying from 526 nm to 800 nm. The calculated diffraction spectrum is shown in Figure S4. The maximum value appears at λ = 899 nm and the full width at half maximum is 318 nm, which can be used as a broadband diffraction grating.

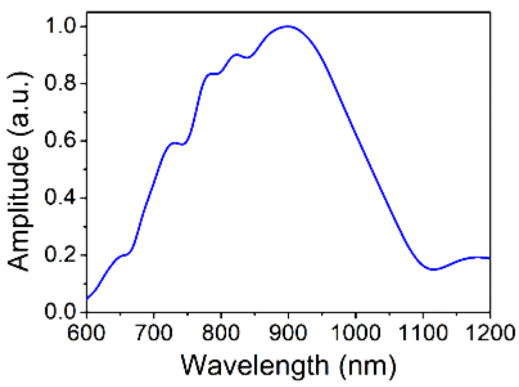

Figure S4: Calculated diffraction spectrum of the chirped grating.

5. Details of the experiment

The spectrum of the light source used in the experiment is shown in Figure S5(a). The far-field angular distribution of the light scattered from the grating at λ = 800 nm was calculated using the FDTD method and is shown in Figure S5(b). In the experiment, the numerical aperture (NA) of the utilized collection objective lens (20×) is NA = 0.25, which has a one-half angular aperture of 14.5°. The collection range of the objective is marked by the white dashed circle, and the collection efficiency of the objective lens is calculated to be 37.3%.

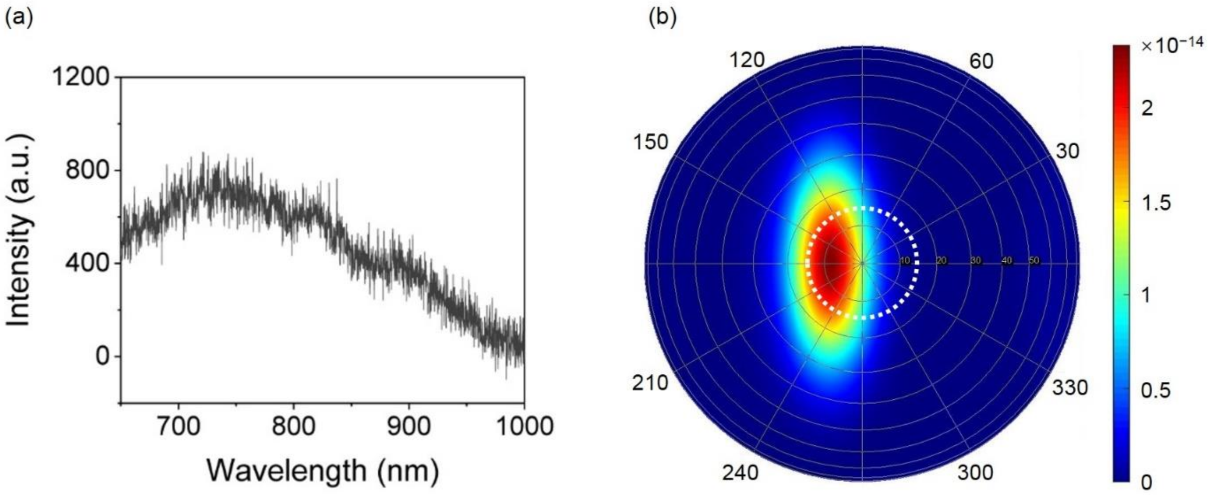

Figure S5: (a) Measured spectrum of the light source. (b) Calculated far-field angular distribution of the light scattered from the grating at λ = 800 nm. The white dashed circle indicates the collection range of the 20× objective.

6. Fitting errors of the polynomial fitting

The root mean squared errors (RMSE) of the polynomial fitting in Figure 3(e) are shown in Figure S6, which shows small errors. The result indicates the reliability of the polynomial fitting. Moreover, the RMSE values are similar for different displacements x_0. Therefore, the fitting errors have limited influences on the determination of extinction wave-lengths.

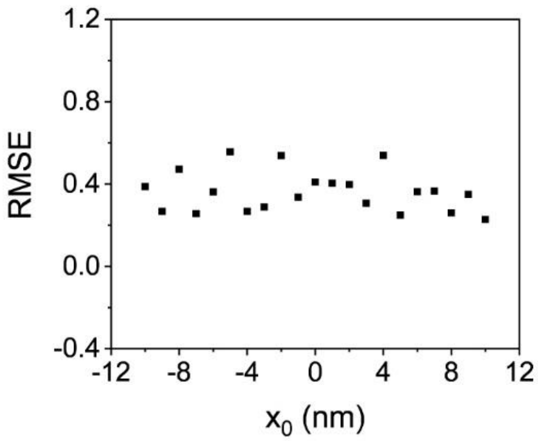

Figure S6: RMSE of the polynomial fitting.

7. Calculated output spectra in linear coordinates for the interference fringes light source

The calculated output spectra in Figure 4(c) were replotted in Figures S7 in linear coordinates, which show that the broadening of the output spectra is similar. The extinction wavelengths were obtained by extracting the wavelengths of the minimum values of the dips in the spectra and the accuracy were not influenced by the broadening of the spectra.

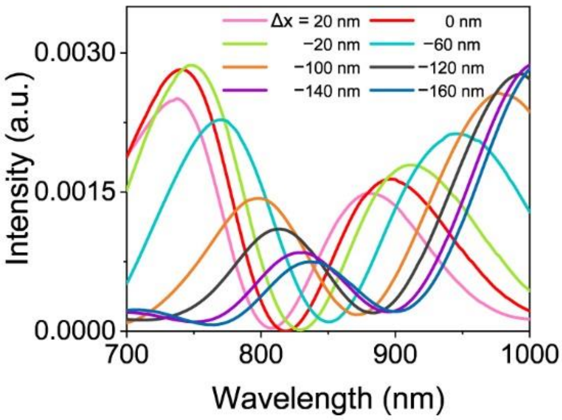

Figure S7: Calculated output spectra for various displacements under the illumination of the interference fringe in linear coordinate.

8. Smoothed curve of the output spectra

With the illumination of the interference fringe, the captured spectra in Figure 5(e) have more than one dip, which are unsuitable for polynomial fitting. Therefore, the data is smoothed by the adjacent-averaging method to eliminate the influence of the spectra oscillations caused by the measurement noise. The smoothed curves of the output spectra for different displacements Δx are shown in Figure S8, and the extinction wavelengths were obtained by extracting the wavelengths of the minimum values of the dips in the spectra.

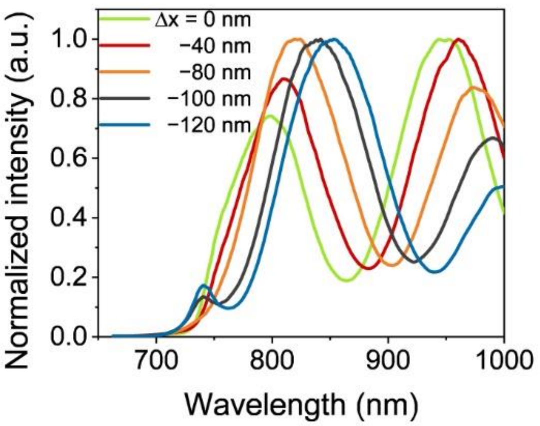

Figure S8: Smoothed curves of the output spectra for different displacements Δ*x*.

9. Influence of incident angle on the interference fringes

To analyze the influence of the incident angle, the calculated spatial intensity distributions of the interference fringes at the incident angle of 30 °and 50 °for λ = 750 nm are shown in Figure S9(a). A larger incident angle results in a smaller full width at half maximum (FWHM) and period.

We calculated the spatial intensity distribution of the interference fringes for λ = 750 and 950 nm at the incident angle of 10 °and show the results in Figure S9(b). Even for a smaller incident angle, the maximum intensity of the fourth-order dispersion fringe at the long wavelength begins to overlap spatially with that of the fifth-order at the short wavelength in the working wavelength range of 750–950 nm.

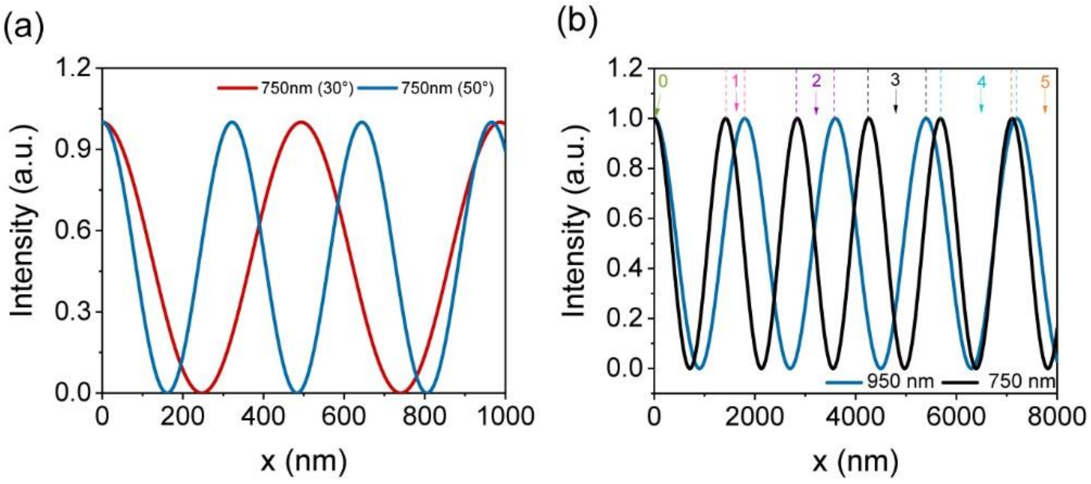

Figure S9: (a) Calculated electric field intensity versus *x* for λ = 750 nm at the incident angle of 30° and 50°. (b) Calculated electric field intensity versus *x* for λ= 750 and 950 nm at the incident angle of 10°. The numbers refer to the interference orders.

10. Design of the two-dimensional displacement sensing by integrating two antenna pair arrays

By integrating two antenna pair arrays, two-dimensional (2D) measurements can be realized. The schematic of the 2D displacement sensor is shown in Figure S10. Two antenna pair arrays with orthognal orientations are integrated. Four white light beams are used to form two interference fringes using the lens L_a and the oil-immersion objective shown in Figure 5(a). When these two interference fringes illuminate on the two antenna pair arrays, 2D displacement detection can be realized.

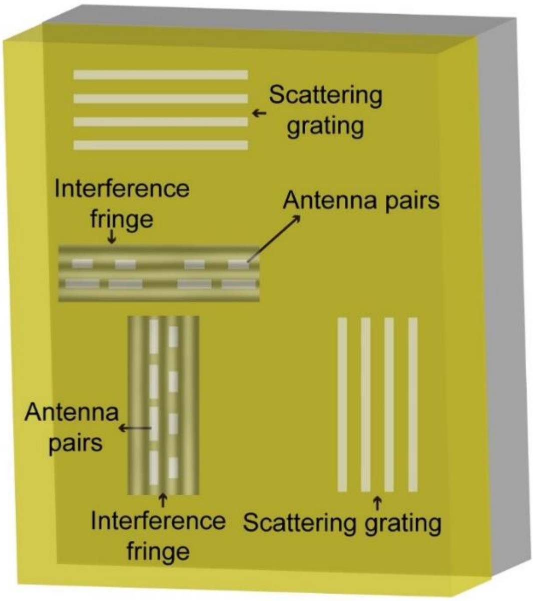

Figure S10: Schematic of the horizontally and vertically distributed antenna pairs for two-dimensional sensing.
